# IL-22 in Atopic Dermatitis

**DOI:** 10.3390/cells13161398

**Published:** 2024-08-22

**Authors:** Julia Laska, Maciej Tota, Julia Łacwik, Łukasz Sędek, Krzysztof Gomułka

**Affiliations:** 1 Student Research Group of Microbiology and Immunology, Department of Microbiology and Immunology, Zabrze, Medical University of Silesia in Katowice, 40-055 Katowice, Poland; 2 Student Research Group of Internal Medicine and Allergology, Clinical Department of Internal Medicine, Pneumology and Allergology, Wroclaw Medical University, 50-369 Wrocław, Poland; 3 Department of Microbiology and Immunology, Zabrze, Medical University of Silesia in Katowice, 40-055 Katowice, Poland; 4 Clinical Department of Internal Medicine, Pneumology and Allergology, Wroclaw Medical University, 50-369 Wrocław, Poland

**Keywords:** interleukin-22, Th22, atopic dermatitis, AD, fezakinumab, wound healing, infections, skin barrier

## Abstract

Atopic dermatitis (AD) is a prevalent and chronic inflammatory skin condition characterized by a multifaceted pathophysiology that gives rise to diverse clinical manifestations. The management of AD remains challenging due to the suboptimal efficacy of existing treatment options. Nonetheless, recent progress in elucidating the underlying mechanisms of the disease has facilitated the identification of new potential therapeutic targets and promising drug candidates. In this review, we summarize the newest data, considering multiple connections between IL-22 and AD. The presence of circulating IL-22 has been found to correlate with the severity of AD and is identified as a critical factor driving the inflammatory response associated with the condition. Elevated levels of IL-22 in patients with AD are correlated with increased proliferation of keratinocytes, alterations in the skin microbiota, and impaired epidermal barrier function. Collectively, these factors contribute to the manifestation of the characteristic symptoms observed in AD.

## 1. Introduction

### 1.1. Definition and Subtypes of Atopic Dermatitis

Atopic dermatitis (AD) is a widespread, chronic disease characterized by intense itching and recurrent eczematous lesions. The condition often begins in infancy and mainly affects children [1]. In a high percentage of cases, AD resolves in childhood. However, in severe cases, it persists into adulthood or begins and recurs later in life [2]. AD is a heterogeneous disorder and, according to recent studies, can be divided into different types in different age and ethnic groups and depending on IgE levels and filaggrin mutation status [3]. There is an IgE-high, extrinsic subtype (with frequent filaggrin mutations), showing allergy to proteins and food allergy, and an IgE-normal, intrinsic subtype, showing allergy to metals. In extrinsic AD, impairment of the skin barrier is observed, while in intrinsic AD, it is preserved. Extrinsic AD is much more common than intrinsic AD, which accounts for about 20% of all cases, with a female predominance. Because of the discovered differences in the phenotype of AD in different populations, a separate division into European (EA) and Asian subtypes has also been proposed [4].

### 1.2. Prevalence

It is estimated that the current prevalence of AD ranges from 2.7% to 20.1% in children and 2.1% to 4.9% in adults [5,6,7]. Multimorbidity among children in a Spanish cohort of 33,591 participants was identified in 43% of the population. The most common chronic comorbid conditions included asthma, affecting 13.1% of the children, psychosocial disorders at a rate of 7.9%, and visual impairment, which was present in 7.8% of the cases [8].

### 1.3. Scoring Atopic Dermatitis (SCORAD)

The SCORAD scale enables the assessment of overall disease activity based on objective and subjective symptoms. Mild AD corresponds to SCORAD levels below 25, and severe AD corresponds to SCORAD levels above 50 [9]. SCORAD consists of three parts—A, B, and C. The first part (A) is the interpretation of the extent of the lesions according to the rule of nines and accounts for 20% of the score. Part B accounts for 60% of the score and assesses the intensity of six items (erythema, swelling/chapping, scratching effect, oozing/shell formation, lichenification, and dryness), each of which is rated on a four-point scale from 0 to 3. Part C comprises subjective symptoms such as itching or insomnia, accounting for 20% of the score. Choosing the most-representative lesion for evaluation, excluding the most severe and the mildest, is essential. A visual analog scale (VAS) ruler assesses subjective symptoms. The distribution of the score is obtained using the formula A/5 + 7B/2 + C. The maximum possible score to be obtained is 103 [10,11]. There is also objective SCORAD, which includes only the objective component and patient-oriented SCORAD [12].

### 1.4. Eczema Area and Severity Index (EASI)

The EASI score integrates body surface area and skin lesion intensity into a single composite score. Because of the scale’s efficiency, EASI has become the most widely used symptom/severity scale in AD research. It first visually identifies the area of disease involvement separately in four areas of the body: head, neck, upper extremities, trunk, and lower extremities, and assigns a score accordingly: 1 (1–9%), 2 (10–29%), 3 (30–49%), 4 (50–69%), 5 (70–89%), and 6 (90–100%). In the next step, the clinician re-evaluates each of the four areas regarding lesion intensity, meaning the presence of erythema, swelling/papulation, exfoliation, or lichenification, assigning an appropriate score from 0 to 3. The score of each area individually is then calculated by multiplying the sum of the region intensity score by the region area score and a region-specific multiplier (different for adults and children). The final score is the sum of the scores of each of the four areas and ranges from 0 to 72 [13]. It is worth mentioning that the EASI assesses only active acute or chronic AD lesions, while the SCORAD also assesses dry skin, pruritus, and insomnia [14].

### 1.5. Investigator Global Assessment (IGA)

The investigator global assessment (IGA) scheme can also assess the AD severity. It classifies patients into separate severity categories based on the description of the lesions [15]. The validated investigator’s general assessment for AD (vIGA-AD) uses a five-point scale:

- A score of 0 = no inflammatory signs of AD present (erythema, induration/papular lesions, lichenization, oozing/scab); post-inflammatory hyper- and/or hypopigmentation may be present;
- A score of 1 = almost invisible erythema, almost invisible induration or papular changes or minimal lichenization; no oozing and no scabbing;
- A score of 2 = slight but apparent erythema (pink), slight but apparent induration/papular lesions, or slight but apparent lichenization; no oozing and no scabbing;
- A score of 3 = prominent erythema (dull red), clearly visible induration/papular lesions, or visible lichenization; oozing and scabbing may be present;
- 4 = significant erythema (dark or bright red), significant induration/papular lesions or significant lichenization; widespread lesions; oozing or scabbing may be present [16].

### 1.6. The Hanifin–Rajka Criteria (HRC)

Hanifin and Rajka’s diagnostic criteria are the most commonly used for diagnosing eczema/AD [17]. The method involves analyzing a total of 27 criteria: 4 primary (pruritus, dermatitis in classical morphology and distribution, chronic/relapsing dermatitis, personal or family history of atopy) and 23 secondary (e.g., ichthyosis/palmar hyperlinearity/keratosis pilaris and facial pallor/facial erythema) criteria [18]. However, this method caused many problems in daily medical practice due to the number of criteria necessary for analysis, four of which required specialized infrastructure [19]. Notably, a patient may not exhibit all of the characteristics required to make a diagnosis at any given time. Also, some of the minor criteria were found to be poorly defined or nonspecific, while others, specific to AD, are relatively rare [20]. For this reason, a British working group has refined the Hanifin and Rajka criteria to improve their practical application and facilitate the work of physicians. Nevertheless, Hanifin and Rajka’s criteria remain valuable in hospital-based settings [21].

### 1.7. Aim of the Study

The aim of this review is to summarize the current understanding of the role of IL-22 in the pathogenesis of AD. In this paper, we assess the impact of IL-22 on skin barrier function, epidermal hyperplasia, and wound healing processes. Moreover, other dermatological conditions, including psoriaris, allergic contact dermatitis, alopecia areata, and rosacea are investigated. We also review studies on novel biological drugs targeting the IL-22 and IL-4/IL-13 axes.

## 2. Pathophysiology of AD

Although the mechanisms underlying AD are largely unknown, several factors are well established, including epidermal barrier disruption, filaggrin deficiency, alterations in cellular immune responses, IgE-mediated hypersensitivity, and environmental factors (airborne formaldehyde, harsh detergents, fragrances, or preservatives). The Th2/Th1 imbalance observed in AD may cause changes in cell-mediated immune responses and promote IgE-mediated hypersensitivity. Furthermore, IL-17- and IL-22-related cytokines have been implicated in leading to skin barrier dysfunction and AD development. Various genetic alterations, e.g., R501X, 2282del4 *filaggrin* mutations, and *SPINK5* loss of function mutation, have also been identified as disrupting skin barrier function, resulting in the picture of AD [22,23,24].

### 2.1. Th2 Response

Th2-type immune responses play an essential role in the pathogenesis of AD. The T helper-2 (Th2) lymphocyte response, with increased production of IL-4, IL-13, or IL-31, is mainly associated with the acute and subacute phases [25,26,27]. Th1 lymphocytes and their cytokines, on the other hand, are associated with the chronic phase [26]. IL-31 is a cytokine involved in the sensation of itching [28]. IL-13 is thought to be the primary effector triggering disease onset, while IL-4 is a critical enhancer of type 2 immunity [29]. The JAK-STAT pathway is mainly involved in the signaling of these cytokines. Studies have shown that topical application of JAK inhibitors attenuates Th2-dependent skin reactions, reduces pruritus, and repairs skin barrier dysfunction [30]. Research has revealed that Th2 lymphocytes and their cytokines cause increased sensitization of transient receptor potential (TRP) cation channels, which play a significant role in neuroinflammation, itching, and pain. These data may contribute to optimizing the treatment of AD [31].

### 2.2. Th17 Response

Th17-response is also related to the pathogenesis of AD. It was found that the IL-17+CD4+ T-cell number was increased in the peripheral blood of AD patients and further correlated with the severity of the disease course. IL-17 produced by Th17 lymphocytes is thought to coordinate local tissue inflammation by increasing the synthesis of proinflammatory and neutrophil mobilizing cytokines and chemokines (e.g., IL-6, GM-CSF, and tumor necrosis factor α—TNF-α or IL-1β) [32]. Th17 also produces other cytokines, for instance, IL-22 and IL-26, which may play an important role in combining Th17 and Th2 responses, leading to the development of AD [33]. The Th17 pathway plays a role in chronic AD, with differences depending on age and ethnicity [25]. Increased Th17 activation compared to adults was also found in pediatric patients [34,35].

### 2.3. Th22 Response

**Figure 1:**
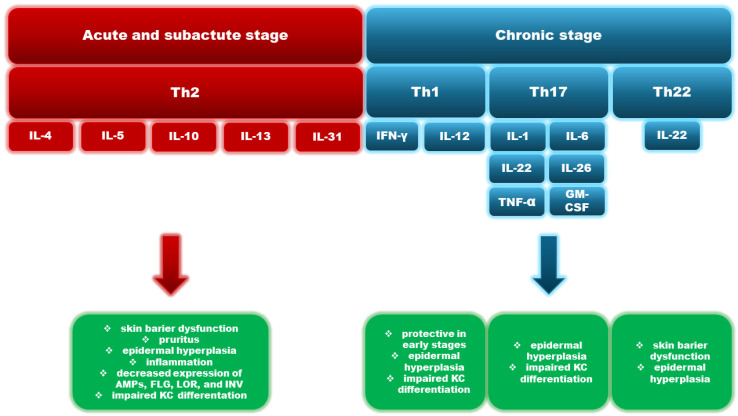
Cytokines in the pathogenesis of atopic dermatitis. KC—keratinocytes; AMPs—antimicrobial peptides; FLG—filaggrin; LOR—loricrin; INV—involucrin.

The activation of Th22 cells predominates mainly in the acute phase of AD, accompanied by a shift in balance towards Th2 lymphocytes [25]. These cells appeared to downregulate terminal differentiation genes and tight junction products such as claudins, contributing skin barrier defects. However, in the chronic phase of AD, Th2 and Th22 responses are also enhanced despite the dominance of the Th1 axis [36]. Th22 is also the source of IL-22, recognized as one of the pathogenic cytokines in AD, the exact role of which will be elaborated later on [37,38]. In addition, Th17 and Th22 expression appeared significant in different ethnic and age groups. Patients with the Asian subtype of AD had significantly higher Th17 and Th22 activation compared to patients with EA, while African American patients appeared to have this activation reduced [39,40] (Figure 1). 

## 3. Review of IL-22 Functions

Interleukin-22 (IL-22) is a proinflammatory cytokine primarily associated with Th17 and Th22 cells. Th17 cells are a subset of CD4+ T cells that produce IL-17 and IL-22, among other cytokines. This cytokine is also produced by other immune cells, including type 3 innate lymphoid cells (ILC3), γδ T cells, NK cells, NKT cells, mucosal-associated invariant T cells (MAIT cells), and dendritic cells (DC) [41]. Th22 cells represent a subset of CD4+ T cells specialized in the exclusive release of IL-22. These cells express the aryl hydrocarbon receptor (AhR) as their key transcription factor. The presence and activation of AhR play a critical role in facilitating the efficient production of IL-22 by Th22 cells. IL-22 production in Th22 cells is also dependent on the retinoic acid receptor (RAR)-related orphan receptor gamma (RORγt) [41,42]. The production of IL-22 depends on cellular stimulation mediated by various cytokines and transcription factors. This interleukin is expressed in response to proinflammatory cytokines released by myeloid cells, such as IL-1β, IL-6, TNFα, and IL-23 [43].

IL-22 is a class II cytokine categorized in the IL-10 family of cytokines due to its biochemical and functional characteristics. This molecule shows 22% homology to mouse IL-10. The IL-22 monomer is composed of six α-helices (labeled A–F) and a small N-terminal helix, forming a compact six-helix bundle cytokine [44,45]. The functional form of IL-22 exists as a monomer, but this molecule can also form dimers and tetramers, which are non-functional storage forms [46]. Structural analysis has unveiled three potential glycosylation sites within helix A, helix C, and the AB loop [44]. Moreover, mass spectroscopy experiments have provided compelling evidence that hexasaccharides, consisting of two N-acetyl glucosamines, three mannoses, and one fucose residue, are indeed covalently linked to the IL-22 protein expressed in *Drosophila melanogaster* [47].

The human *IL-22* gene is situated on chromosome 12q15, near the genes responsible for encoding IFN-γ and IL-26, both of which belong to the *IL-10* gene family. The *IL-22* gene comprises five exons, and its open reading frame spans 537 base pairs, resulting in the translation of a 179 amino acid protein [48]. Notably, the secreted form of IL-22 typically consists of 146 amino acids [49]. Research investigations have identified the constitutive expression of IL-22 in the thymus and brain. Conversely, induced expression has been observed in the lung, skin, pancreas, gut, liver, and spleen [50].

### 3.1. IL-22 Receptor

IL-22 utilizes its receptor complex to trigger downstream effector functions. The IL-22 receptor is composed of the IL-22R1 and IL-10R2 subunits. It contains three main domains: the extracellular, transmembrane, and intracellular signaling region [51,52,53,54]. IL-10R2 is expressed in a wide range of cells, including immune cells, whereas IL-22R1 expression is thought to be limited to epithelial, intestinal epithelium, renal tubular, and pancreatic ductal cells [55,56,57]. In the skin, IL-22R is expressed by keratinocytes and dermal fibroblasts [58].

IL-22 binds to the IL-22R1 subunit, which undergoes a conformational change that allows the binding of IL-10R2, initiating the downstream signaling cascade [59]. Binding IL-22 to the receptor induces several downstream cascades, including activation of Janus kinase 1 (JAK1) and tyrosine kinase 2 (Tyk2), which further phosphorylates and activates signal transducer and activator of transcription 3 (STAT3), STAT1, or STAT5 which are responsible for a broad spectrum of downstream effects. Moreover, the IL-22/IL-22R complex activates MAP kinase pathways such as the extracellular signal-regulated kinase (MEK/ERK), c-Jun N-terminal kinase (JNK), and the p38 pathways, thereby mediating IL-22 in eliciting its effector functions [52,60,61,62].

### 3.2. IL-22 Binding Protein

**Figure 2:**
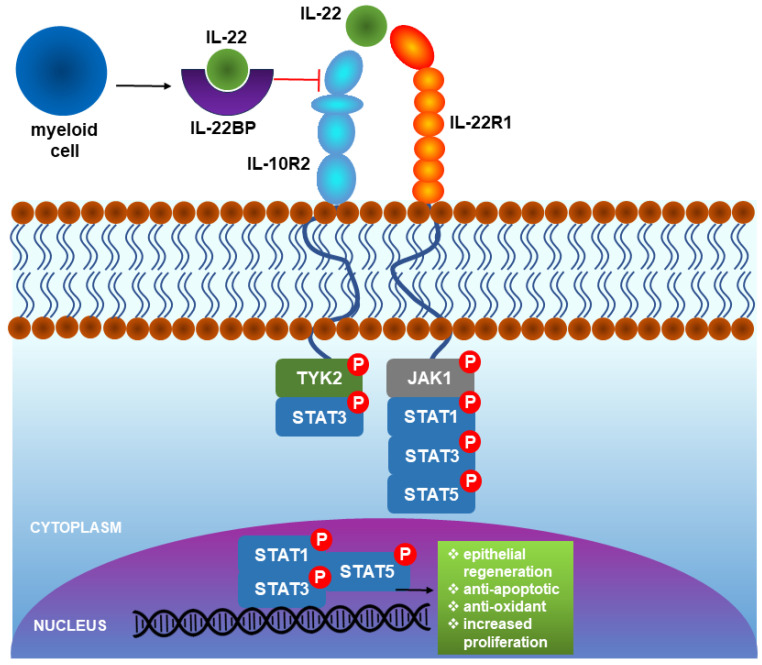
The mechanism involving IL-22, the IL-22 receptor, and IL-22 binding protein (IL-22BP) operates through the presented signaling pathways. The IL-22 receptor is composed of two subunits: IL-10R2 and IL-22R1. The interaction among IL-22, the IL-22 receptor, and IL-22BP occurs within the extracellular domain, while the signaling pathways occur within the cytosol and nucleus.

The activity of secreted IL-22 is regulated by an inhibitor known as IL-22 binding protein (IL-22BP or IL-22RA2), which acts as a natural antagonist of IL-22. IL-22BP is a homolog of IL-22RA1 primarily produced by myeloid cells [63,64]. IL-22BP shares 34% sequence homology with the extracellular domain of IL-22R. The shared homology between IL-22BP and IL-22RA1 extends to the secondary and tertiary structures. This structural similarity enables IL-22BP to bind to IL-22 in a manner that interferes with the binding of IL-22 to its receptor. IL-22BP plays an essential role in controlling the biological activity of IL-22 in healthy individuals and during infection or chronic inflammatory diseases (Figure 2) [65,66].

## 4. IL-22 in Wound Healing Processes

Wound healing is a complex and highly coordinated process utilizing keratinocytes, fibroblasts, and immune cells mediated by several cytokines, chemokines, and growth factors to restore the epidermal barrier and tissue architecture after acute injury. IL-22 plays a central role in this process, stimulating the proliferation, migration, and differentiation of the cells involved in tissue repair [67,68,69]. The modulation of this molecule, whether through its up-regulation or down-regulation, results in a diverse range of outcomes that characterize its biological and pathological effects [52,60]. For this reason, disruptions in the activity of IL-22 can lead to chronic inflammatory diseases, impaired wound healing, keloid scars, infections, and carcinogenesis [52,67,70,71].

Keratinocytes play a crucial role in forming a functional skin barrier and defending against various environmental microbes and chemicals. The balance between keratinocyte proliferation and apoptosis is essential for maintaining skin homeostasis [72]. IL-22 impacts the functions of keratinocytes by inhibiting the production of several proteins crucial for the terminal differentiation of keratinocytes, including keratin 1 and 10, profilaggrin, loricrin, and desmocollin [41,73]. Upon skin inflammation, the upregulation of IL-22 expression facilitates the proliferation and migration of keratinocytes towards the injury site. Meanwhile, during the wound healing processes, IL-22 suppresses keratinocyte differentiation [72,74].

IL-22 has been identified as a factor that promotes communication between immune cells and fibroblasts during skin wound healing. In a mouse wound healing model, fibroblast function was shown to be IL-22-dependent upon acute injury, and this cytokine was identified as a critical mediator for normal extracellular matrix protein (ECM) production and myofibroblast differentiation during the healing processes [74,75].

In a particular study, IL-22(−/−)mice exhibited significant abnormalities within the dermal layer of the skin following full-thickness wounds [75]. The absence of IL-22 during an injury has been observed to lead to compromised granulation, ECM production, tissue formation, and hindered wound contraction. Primary dermal fibroblasts express IL-22R1, which, upon binding with IL-22, triggers the downstream JAK/STAT pathway. Subsequent dimerization and translocation of STAT molecules into the nucleus result in an increased production of ECM components, including fibronectin and collagen. Therefore, in murine models deficient in IL-22, defective wound contraction and reduced ECM production are evident. A reduced population of myofibroblasts in the wound can result in insufficient wound contraction and impaired ECM formation, as observed in IL-22(−/−)mice [68].

The IL-22/IL-22R complex plays a crucial role in maintaining the integrity of the skin epithelium by promoting the proliferation and survival of epithelial cells. Hence, it contributes to protecting the mucosal barrier following tissue damage. Its malfunction leads to abnormal epidermal proliferation [33,70,71,76]. Skin-resident commensal microbes produce antimicrobial peptides (AMPs), which not only increase the regular AMP production by keratinocytes but also play a beneficial role in maintaining inflammatory balance by suppressing excessive cytokine release following minor epidermal injuries. IL-22 enhances AMP production together with IL-17 and induces epidermal proliferation [23,33]. AMPs, which are a key part of host defense, e.g., β-defensins 2/3, SA1007, 1008, 1009, and lipocalin-2, can kill or inhibit the growth of microbes, representing an essential aspect of barrier protection. Specialized epithelial cells called Paneth cells are the primary source of AMPs in the intestine. AMPs are also produced by keratinocytes [43,77]. IL-22 plays a crucial role in protecting against infections by promoting the increased production of AMPs, thereby enhancing antibacterial competence [45,60]. It is hypothesized that variations and changes in the skin microbiome concerning AD status are linked to the secretion of bacteriocins and AMPs by commensal bacteria [23].

Furthermore, research has revealed that IL-22 stimulates the expression of anti-apoptotic genes such as *Bcl-2* and *Bcl-xL*, as well as matrix metalloproteinases such as MMP1/3, thereby enhancing cell proliferation and contributing to the remodeling of the epidermis and tissue repair mechanisms [41]. IL-22 has been shown to improve cell viability in human epidermal keratinocytes (HEKs). It inhibited apoptosis induced by TNF-α and IFN-γ in HEKs, as IL-22 upregulated Bcl-xL while downregulating Bax production when TNF-α and IFN-γ were present [76].

However, the regenerative cell survival signaling of IL-22 has the significant potential to shift toward tumor formation when overactivated in an uncontrolled manner. In certain cases, IL-22 may indeed contribute to tumor cell proliferation, survival, and invasion [71,78,79]. Pathological wound healing can be expressed by either overproduction of scar tissue, resulting in keloid scars and adhesions, or failure to heal, resulting in persistent chronic wounds. Higher levels of IL-22 were observed in keloid scars than in normal scars [67,80].

In summary, IL-22 is a cytokine that plays a critical role in skin homeostasis and wound healing. Consequently, the dysregulation of IL-22 and immune cells that produce IL-22 is linked to various inflammatory skin conditions, including AD.

## 5. IL-22 Protects against Infections

IL-22 is pivotal in generating innate immune responses against various infections. IL-22 is an important cytokine that maintains homeostasis at barrier surfaces. In response to epithelial damage or dysfunction, immune cells are activated to produce IL-22, involved in the repair and protection of skin and mucosa [81,82].

Mucin is one of the mucus components, which is produced by specialized epithelial cells called goblet cells. In mucosal tissues, mucus constitutes the first line of defense against invading pathogens. The mucus layer in certain epithelial cells serves as an innate mechanism against bacterial and viral infections and also for helminths and protozoan parasites [83,84,85]. IL-22 induces the expression of *mucin* genes in mucosal epithelial cells. This cytokine serves to upregulate several mucus-associated genes. IL-22-induced JAK/STAT signaling was implicated in regulating *MUC1* and *MUC13* expression. Also, expression of the *MUC2* gene was elevated upon the addition of IL-22 [86,87,88,89].

Impaired IL-22 production by group 3 innate lymphoid cells (ILC3) reduces the expression of IL-22-dependent antimicrobial peptides, RegIIIβ and RegIIIγ. Reg-IIIγ exhibits antibacterial activity against Gram-positive bacteria by recognizing peptidoglycan, inducing damage to the bacterial cell wall and triggering cytoplasmic leakage. Additionally, Reg-IIIγ is essential for establishing spatial segregation, contributing to the separation of the microbiota from the surface of the host’s small intestinal epithelium [82,83,84].

Studies on *Citrobacter rodentium* and *Escherichia coli* have demonstrated that IL-22 suppresses the systemic growth of bacteria. This was shown by restricting iron availability to the pathogen through the production of hemopexin [85,90]. IL-22 has been demonstrated to enhance epithelial integrity and facilitate tissue repair at barrier surfaces, particularly in the lungs, following inflammation, as type 3 innate lymphoid cells (ILC3s) are constitutively present in lung tissue and can rapidly produce IL-17 and IL-22 upon stimulation. During respiratory infection caused by *Streptococcus pneumoniae*, IL22RA2(−/−) mice were more resistant to contagion, had increased IL-22 levels in lung tissues, and sustained longer survival upon infection than control mice [91,92]. The skin of individuals with AD exhibits reduced bacterial diversity, characterized by an elevated presence of *Staphylococcus* spp. and *Corynebacterium* spp. [93,94]. *Staphylococcus aureus* colonizes the skin affected by AD and is crucial in initiating and exacerbating AD [95]. *Staphylococcus aureus* can increase the expression of proinflammatory cytokines such as TSLP, IL-4, IL-12, and IL-22. Moreover, it can induce mast cell degranulation, leading to an exaggerated Th2 immune response. This shift toward a Th2-type response contributes to increased inflammation, allergic reactions, and the distinctive symptoms associated with conditions like AD [23].

In the context of both viral and bacterial pneumonia, it has been noted that activated ILC3s release the cytokines IL-17 and IL-22. This cytokine secretion is crucial in bolstering microbial clearance and facilitating tissue repair. The risk of developing severe complications from an influenza virus infection is increased in patients with chronic AD. The compromised barrier function observed in influenza infection has been linked to decreased claudin expression within epithelial cells, resulting in impaired formation of tight junctions. In the keratinocytes of the stratum corneum, claudin helps to maintain the skin barrier, protecting against water loss and pathogen penetration [92,96].

Enteropathogens damage the intestinal epithelium, induce the release of proinflammatory cytokines, and cause diarrhea. IL-22-mediated antibacterial activity against *Salmonella enterica Typhimurium* is executed through phagolysosomal fusion. It also activates the STAT3 signal pathway related to suppressing apoptosis in intestinal epithelial cells to maintain intestinal epithelial barrier function and prevent infection [97,98]. Furthermore, IL-22 stimulation leads to the secretion of epithelial claudin-2, increasing tight junction permeability to Na^+^ ions and water, which results in diarrhea and enteric pathogen clearance [99,100]. Moreover, mice lacking claudin 1 were observed to succumb to wrinkled skin and profound dehydration. This study indicated the indispensable role of claudin in maintaining skin barrier function [23].

IL-22 and IL-17 are crucial natural defense mechanisms against chronic mucocutaneous candidiasis (CMC). CMC, including oral CMC, represents the initial manifestation of autoimmune polyendocrine syndrome type 1 (APS1) in 70–80% of cases. This presentation is characterized by the presence of autoantibodies targeting IL-17A, IL-17F, and IL-22 [101,102,103]. Several studies suggest that IL-22 helps control oral candidiasis. Mice with impaired IL-22 function exhibit susceptibility to oropharyngeal candidiasis (OPC), and decreased IL-22 expression is linked to CMC in humans [104,105].

Hepatitis B virus (HBV) and hepatitis C virus (HCV) are one of the most critical risk factors for liver cancer [106]. The expression of IL-22 is increased in individuals with chronic HBV and HCV infections [107]. This cytokine exhibits two action profiles in liver diseases: a hepatoprotective effect in acute liver injury and a proinflammatory effect in other disease models [108,109]. The inflammatory immune response triggered by HBV infection plays a crucial role in the development of hepatocellular carcinoma (HCC). Studies have indicated that elevated levels of serum IL-22 and a high presence of IL-22-producing cells infiltrating tumors are unfavorable prognostic indicators for patients with HCC and are strongly associated with reduced overall survival [109,110].

## 6. IL-22 Promotes Epidermal Hyperplasia and Skin Barrier Dysfunction in AD

The epidermis comprises epithelial cells, immune cells, and microbes, forming a robust physical and functional barrier that safeguards human skin. The most notable pathological observations in the skin of individuals with AD are related to defects in the skin barrier. The epidermal barrier-related terminal differentiation, tight junctions, lipid biosynthesis, and metabolism markers are all downregulated in AD. Various factors, such as immune dysregulation, filaggrin mutations, insufficiency of antimicrobial peptides, and imbalances in skin microbiota, collectively contribute to these skin barrier abnormalities [111,112,113].

Initiation of AD is preceded by an acute phase characterized by increased activation of Th2, Th22, and Th17 lymphocytes. The progression to the chronic phase is characterized by the concurrent activation of Th1 cells along with the persistent activation of Th2 and Th22 cells. There is mounting evidence indicating a significant upregulation of IL-22 in the skin of individuals with AD [30,114,115]. IL-22 has been associated with epidermal hyperplasia and has been implicated in inhibiting the terminal differentiation of keratinocytes. Additionally, it induces processes such as the expression of S100A120 and S100A136, leading to hyperplasia [115,116,117]. The expression of IL-22 is also associated with markers such as keratin 6 and keratin 16, which serve as indicators of epidermal growth and proliferation [23].

The sensation of atopic itch is mediated by the interplay between epidermal barrier dysfunction, upregulated immune cascades, and the activation of structures in the central nervous system [118]. Gastrin-releasing peptide receptor (GRPR) has been regarded as a highly versatile receptor in the spinal cord that was activated by gastrin-releasing peptide (GRP) in dorsal root ganglion neurons to mediate the non-histaminergic itch and pathological itch conditions principally [119,120]. Within the spinal cord, itch signals are transmitted through the spinothalamic tract via GRPR+ neurons [121]. The presence of GRP+ cells in the skin correlated with both AD and pruritus severity. IL-22 triggered the upregulation of GRP in dermal cells, dermal afferent fibers, and skin-innervating ganglion neurons. This increased expression demonstrated a positive correlation between the intensity of itching and the frequency of scratching behaviors. Additionally, IL-22 induced a distinct elevation in the expression of the GRP receptor on keratinocytes in AD skin [117].

## 7. IL-22 in Other Dermatological Diseases

### 7.1. Psoriasis

The hallmark histological characteristics of psoriatic skin comprise epidermal thickening (acanthosis), keratinocyte hyperproliferation (hyperkeratosis), and the infiltration of immune cells within both the dermis and epidermis. Psoriasis vulgaris is the most common type. It is typically diagnosed by its distinctive red plaques with well-defined edges and silvery-white scales, commonly found on the elbows, knees, scalp, and lower back. However, it can also be more widespread [122]. In the early stages of psoriasis, a TLR7 agonist activates plasmacytoid dendritic cells (pDCs) to produce interferons (IFNs). LL37, a peptide derived from cathelicidin, plays a crucial role in initiating lesions by binding to nucleic acids and stimulating pDCs to release IFN-α/β. LL37/RNA complexes also activate resident myeloid DCs, leading to the production of IL-12 and IL-23, which are central cytokines in psoriasis. In chronic disease, the primary pathogenic pathway involves mature dermal and inflammatory myeloid DCs producing IL-23 and IL-12. These cytokines activate Th17, Th1, and Th22 cells, which further contribute to the inflammatory cytokine environment and affect keratinocytes. Keratinocytes then produce chemokines and AMPs that amplify cutaneous immune responses, perpetuating the inflammatory cycle [122].

Psoriasis arises from an imbalance in immune cell regulation and cytokine secretion in the skin. Notably, the activation of Th17 cells and the cytokine IL-17 are considered primary contributors to the pathogenesis of psoriasis [123]. Furthermore, research has indicated that cells producing IL-22 and the dysregulation of IL-22 levels play a significant role in the development of psoriasis. Patients diagnosed with psoriasis exhibited markedly elevated levels of IL-22 compared to those observed in healthy individuals [124]. Consequently, increased IL-22 expression levels have been observed in the skin and peripheral blood of patients with psoriasis compared to healthy individuals. Interestingly, notable disparities in the expression of IL-17 and IL-22 were identified when comparing pediatric psoriasis patients to both pediatric healthy controls and adult psoriasis patients. The involvement of IL-22 is significant in the pathogenesis of pediatric psoriasis, suggesting that it could serve as a unique therapeutic target specifically for this population [125].

### 7.2. Allergic Contact Dermatitis

Allergic contact dermatitis (ACD) can be significantly debilitating. It is characterized by an intensely pruritic erythema, edema, and frequently the presence of vesicles at the locations where allergens come into contact with the skin. Elevated serum levels of IL-22 have been documented in patients with ACD to nickel [126]. Furthermore, a significant infiltration of IL-22-secreting T cells is observed in the skin following re-exposure to nickel [127,128]. In a study by Robb et al., both T-cell cultures and in vivo sensitization of mice with haptens were employed to evaluate the role of prostaglandin E2 (PGE2) in the production of IL-22. In response to antigen or hapten sensitization, keratinocytes become activated and produce pro-inflammatory cytokines. Meanwhile, resident skin immune cells, such as dendritic cells, capture these antigens and migrate to secondary lymphoid organs. There, they present the antigens to T lymphocytes, leading to their activation and differentiation into effector T cells. This process triggers ACD, when the antigen or hapten is repeatedly applied to the skin. Traditionally, both type 1 and type 2 helper T cells were believed to play a role in the pathogenesis of ACD. However, recent research has revealed that IL-17 and IL-22, produced by Th17 and other activated T cells such as Th22 cells, are also crucial in driving the initiation and progression of ACD. The authors discovered that PGE2 plays a pivotal role in advancing ACD by promoting IL-22 production via T cells [129].

### 7.3. Alopecia Areata

Alopecia areata is a common immune-mediated condition that causes non-scarring hair loss, characterized by circular patches on the scalp or any hair-bearing area. It is classified based on the extent of hair loss: patchy alopecia areata (partial scalp hair loss), alopecia totalis (complete scalp hair loss), and alopecia universalis (complete loss of scalp and body hair). Th1, Th2, and Th17 activation has been shown to be involved in the development of this disease. Most studies found IL-22 levels in alopecia areata patients comparable to healthy controls, though Atwa et al. observed an increase [130,131,132]. Further research is needed.

### 7.4. Rosacea

Rosacea is a common chronic inflammatory disorder that primarily affects the centro-facial skin. It is characterized by persistent facial erythema, telangiectasia (visible blood vessels), and a range of inflammatory lesions, including papules, pustules, and swelling. Additional clinical manifestations may include transient erythema, flushing, ocular involvement, and, in some cases, phymata. The key feature of rosacea is persistent facial redness [133]. Studies have shown that in acute rosacea, there is an overproduction of type I interferons, which correlates with an increase in plasmacytoid dendritic cells in skin lesions. Commensal skin bacteria are required for pDC activation and type I IFN production, but in rosacea, this process is exacerbated by harmful bacteria and antimicrobial peptides. Type I IFNs then trigger an immune response that increases IL-22 levels and makes endothelial cells more responsive to IL-22. This connection links skin dysbiosis to neoangiogenesis, a key feature of rosacea [134]. Despite recent advances, the pathomechanisms of rosacea are still unclear, and treatments remain broad and symptomatic.

## 8. Novel Drugs in the Treatment of AD

### 8.1. Biological Drugs

#### 8.1.1. Fezakinumab (FZ, ILV-094)

Increased secretion of the IL-22 cytokine is characteristic of AD and plays an important pathogenic role in disease initiation [135]. FZ (ILV-094) is a human IgG1-lambda monoclonal antibody that directly binds to IL-22 and prevents the formation of the IL-22/IL-22 receptor complex (IL-22R1). Subsequently, the binding of this binary complex to the extracellular domain of the interleukin-10 receptor 2 (IL-10R2) inhibits downstream signaling through this receptor [136].

A randomized, double-blind, placebo-controlled study was performed, proving significant efficacy and tolerability of FZ in the treatment of AD. Sixty patients with moderate-to-severe AD were selected for the study; a randomized group of 40 patients received the anti-IL-22 antibody intravenously every two weeks for 10 weeks, with a follow-up assessment until week 20. Treatment outcomes were assessed by comparing the change in SCORAD score to the baseline. As early as the 4th week of the experiment, patients receiving FZ showed a permanent and more significant mean decrease in SCORAD baseline as compared to the placebo group. This difference persisted throughout the 10-week treatment period until the end of the study at week 20. More noticeable differences were observed, particularly in patients with severe AD [137].

The same researchers also evaluated the cellular and molecular effects of IL-22 blockade in the tissues of patients with moderate-to-severe AD. They examined skin biopsy samples of patients with and without lesions from the patients treated with FZ (*n* = 39) and compared them to the placebo group (*n* = 20). Biopsy samples were taken before treatment (baseline/week 0), during treatment (week 4), and after treatment (week 12). Transcriptomic and immunohistochemical methods were used to analyze the samples. The results showed a greater reversal of the genomic profile of AD as a result of taking FZ than patients taking a placebo. It was also confirmed that treatment effects were dependent on high baseline IL-22 expression, and these patients exhibited significantly higher mean transcriptomic improvement and response to FZ treatment. This group was also characterized by significant suppression involving genes representing mediators of general inflammation (MMP12), T-cell activation (ICOS and CD86/CD28), innate immune responses (MX1 and CXCL8) and molecules related to Th1 lymphocytes (IRF1, CXCL9, CXCL10, and CXCL11), Th2 lymphocytes (CCL13, CCL17, CCL18, and CCL22), Th17 lymphocytes (CCL20, PI3/elaphin, CXCL1, and IL36G), and activation of Th17/Th22 lymphocytes (S100A7, S100A8, and S100A12) [138]. In addition, treatment with FZ increased the expression of IL-34 and IL-37, which are negative immune regulators [138,139]. The researchers also identified predictive markers of response to FZ, the most important of which were genes involved in the activation and differentiation of T lymphocytes and dendritic cells [138]. Surprisingly, some studies reported that even though promising effects were obtained in clinical trials with some biological drugs, several patients have been identified as having a poor response to treatment.

Badi et al. attempted to determine whether the disease signature identified in AD is evident in adults with severe asthma and whether the transcriptomic signature for AD patients who respond clinically to FZ is enriched in severe asthma [140]. This study determined the AD signature via differential analysis of expressed genes between skin biopsies containing lesions and those without lesions. The FZ response signature was obtained before and after 12 weeks of treatment. Subsequently, the researchers used gene set variation analysis to obtain enrichment scores for AD and AD–FZ response signatures in their Unbiased Biomarkers for the Prediction of Respiratory Disease Outcomes asthma cohort. The AD disease signature, including inflammatory pathways, T cells, TH2, and TH17/TH22, was enriched in the blood and sputum of patients with asthma of increasing severity. Furthermore, the AD gene signature became most apparent in patients characterized as neutrophilic or mixed granulocytic subtypes, as did the FZ response signature. The analysis showed that IL-22 mRNA expression did not correlate with FZ response signatures in the blood, sputum, bronchial, and nasal brushings. Simultaneously, there was a significant correlation between sputum IL-22 protein and the Th22/IL-22 gene signatures in the sputum, nasal, and bronchial brushings. The researchers suggest that this may reflect local expression of IL-22 protein in the airways, which is not detected at the mRNA level or not observed due to the lack of proteomic data for bronchial and nasal brushings. However, the increased FZ-response signature suggests a significant effect of IL-22 on downstream signaling. IL-22 mRNA in tissues did not correlate with FZ response enrichment scores, but this response signature was associated with TH22/IL-22 pathways. This study concluded that the FZ response signature in AD identifies patients with severe neutrophilic asthma as potentially responsive to FZ therapy [141].

Notably, side effects associated with the use of FZ are often upper respiratory tract infections caused by different pathogens, including viruses and fungi [142]. IL-22, among other functions, is known to play a crucial role in the first line of defense in candidiasis [143]. Studies in IL-22-deficient mice have shown that they are susceptible to oropharyngeal candidiasis, and reduced IL-22 expression is associated with chronic mucocutaneous candidiasis in humans [104]. Taking all this into account, special attention should be paid to the possible association of FZ with candida infections.

#### 8.1.2. Dupilumab

Dupilumab is the only biologic drug approved to date by the U.S. Food and Drug Administration for the treatment of moderate-to-severe AD in adults and children [144]. It works by blocking IL-4/IL-13 signaling and inhibiting receptor signaling. Hence, dupilumab affects three primary mechanisms in the disease: reduced skin barrier function, IgE class switching, and TH2 differentiation [145]. The administration of dupilumab resulted in substantial improvements in EASI and VAS scores. Additionally, it led to a reduction in serum levels of thymus and activation-regulated chemokine (TARC), IgE, and IL-22. However, the serum levels of IL-13 and IL-31, as well as the number of intraepidermal nerve fibers (IENFs), remained unchanged. Notably, there was an increase in the serum levels of IL-4. Studies have shown that, compared to placebo, it significantly improves the clinical signs and symptoms of AD, is well tolerated, and shifts the lesion transcriptome toward a lesion-free phenotype [116].

#### 8.1.3. Tralokinumab

A promising drug is tralokinumab, a monoclonal antibody with a high affinity for IL-13, to which it binds explicitly and inhibits it [146]. Phase III studies in which patients were administered subcutaneous tralokinumab, or placebo, at a dose of 300 mg every 2 weeks showed that tralokinumab monotherapy was superior to placebo at week 16 and was well tolerated until week 52 of treatment [147]. The efficacy and safety of tralokinumab in combination with topical corticosteroids (TCS) in patients with moderate-to-severe AD was also evaluated. A double-blind, placebo- and TCS-controlled phase III study showed that tralokinumab 300 mg in combination with TCS when needed was effective and well tolerated [148,149]. In another study, a significant alteration in AD biomarkers associated with inflammation through the Th2 pathway was found after tralokinumab treatment, evidenced by changes in CCL-11 and CCL-17 levels, as well as through the Th17/Th22 pathways, indicated by a decrease in IL-22, S100As, DEFB4, and IL36G levels [150].

#### 8.1.4. Lebrikizumab

Another potential drug for AD is lebrikizumab, a monoclonal IL-13 inhibitor [151]. Two randomized, double-blind, placebo-controlled phase 3 studies among patients with moderate-to-severe AD found lebrikizumab treatment to be effective after 16 weeks of study [152].

#### 8.1.5. Tezepelumab (AMG-157/MEDI9929)

Tezepelumab (AMG-157/MEDI9929) is a human monoclonal antibody directed against circulating thymic stromal lymphopoietin (TSLP), which plays a pathological role in AD pathophysiology [153]. In 2018, a study was conducted to assess the efficacy and safety of the drug in adults with moderate-to-severe AD. Some patients received tezepelumab and TCS (topical class 3 corticosteroids), while others received placebo and TCS. The study showed a numerical improvement over placebo; however, this was not statistically significant [154].

#### 8.1.6. Nemolizumab

Another monoclonal antibody used in the treatment of AD is nemolizumab. It blocks the α subunit of the IL-31 receptor, modulating the neuroimmune response and rapidly relieving itching [155]. A 16-week study showed that subcutaneous administration of nemolizumab in AD patients resulted in a greater reduction in pruritus than placebo and topical medications. However, further studies are needed to establish its efficacy and safety [156].

#### 8.1.7. GBR 830

GBR 830 is a monoclonal antibody that blocks OX40 (CD134) [157]. It inhibits OX40–OX40L binding, reducing the severity of inflammatory responses, chronicity, and loss of tolerance. A phase 2a study investigating the safety and efficacy of GBR 830 showed that the administration of the drug 4 weeks apart was well tolerated and that a progressive improvement in tissue lesions was achieved [158].

#### 8.1.8. Janus Kinase Inhibitors (JAKi)

Another remarkable group of drugs in the treatment of AD is the Janus kinase inhibitors (JAKi). The Janus kinase family of enzymes plays a key role in immune responses. Receptors for cytokines involved in AD, including IL-22, require the action of JAK [159]. These drugs help achieve significant improvement in symptoms with a low incidence of adverse events. In particular, oral JAK inhibitors, such as aricitinib, abrocitinib, and upadacitinib, have the potential to improve pruritus and skin symptoms rapidly. Examples of other JAK inhibitors include dergocytinib or ruxolitinib [30]. Side effects of oral JAK inhibitors have been mostly mild to moderate and have included acne, nausea, headache, upper respiratory tract infection, herpes infections, and selected laboratory abnormalities [160].

### 8.2. Combined Biological Therapy for AD

Miyano et al. conducted a study attempting to explain the pathophysiological basis of the variability in drug response in individual patients. The researchers conducted a meta-analysis of recent clinical trials of biologic drugs for AD. They developed a mathematical model that reproduces the reported clinical efficacy of nine biologic drugs (dupilumab, lebrikizumab, tralokinumab, secukinumab, FZ, nemolizumab, tezepelumab, anti-OX40 (GBR 830), and recombinant interferon-gamma), describing the pathogenesis of AD at the system level. Next, they simulated the clinical efficacy of hypothetical therapies in virtual patients. Simulations showed that none of the nine drugs showed a clinical response in virtual patients responding poorly to dupilumab, a monoclonal antibody that inhibits IL-4 and IL-13 signaling. Another important finding is that simultaneous inhibition of IL-22 and IL-13 may become a promising drug therapy for patients responding poorly to dupilumab. Inhibition of these two cytokines showed the highest clinical response of all combinations of the two cytokines, with an EASI-75 after 24 weeks of 21.6% (Table 1) [161].

## 9. Conclusions

IL-22 is increasingly recognized as a pivotal cytokine in the pathogenesis of AD. Elevated levels of IL-22 in AD patients are associated with increased keratinocyte proliferation, modifications in skin microbiota, and compromised epidermal barrier function, all of which contribute to the hallmark symptoms of AD. Therapeutic interventions targeting IL-22 and its signaling pathways are emerging as promising strategies for managing AD, offering potential benefits in reducing inflammation and enhancing skin barrier function. Further research on the IL-22 axis is required for the development of personalized therapeutic approaches.

## Figures and Tables

**Table 1:** Characteristics of drugs targeting the IL-22 and IL-4/IL-13 axes in AD.

Drug	Mechanism of Action	Dose	Response to Treatment	Ref.
Fezakinumab	binds to IL-22 and prevents the formation of the IL-22/IL-22 receptor complex (IL-22R1)	loading dose of 600 mg at baseline (day 0), followed by 300 mg at weeks 2, 4, 6, 8, and 10 (last dose)	decrease in SCORAD baseline as compared to the placebo group; reversal of the genomic profile of AD; significant suppression involving genes representing mediators of general inflammation, T-cell activation, innate immune responses and molecules related to Th1 lymphocytes, Th17 lymphocytes, and activation of Th17/Th22 lymphocytes	[140,141]
Dupilumab	blocks IL-4/IL-13 signaling and inhibits receptor signaling	weekly subcutaneous injections of 200 mg of dupilumab after a 400 mg loading dose on day 1	improvement of symptoms of AD; improvements in AD transcriptome; reduced expression of genes involved in type 2 inflammation, epidermal hyperplasia, T cells, dendritic cells, TH17/TH22 activity, and lesional epidermal thickness; increased expression of epidermal differentiation, barrier, and lipid metabolism genes	[116,145]
Tralokinumab	binds to IL-13 and inhibits it	300 mg every 2 weeks	improvement in pruritus, sleep interference, DLQI, SCORAD, and POEM were observed from the first post-baseline measurements	[147,148,149]
Lebrikizumab	binds to IL-13 and inhibits it	250 mg every 2 weeks (loading dose of 500 mg at baseline and week 2)	a higher percentage of patients had an EASI-75 response in the lebrikizumab group than in the placebo group; reduced itch and itch interference with sleep	[152]
Tezepelumab	anti-TSLP antibody that prevents TSLP–TSLPR interactions	280 mg every 2 weeks plus class 3 TCS	a higher percentage of patients achieved EASI-50 versus placebo plus TCS	[154]
Nemolizumab	blocks the α subunit of the IL-31 receptor (IL-31α)	60 mg	reduction in pruritus	[155,156]
GBR 830	blocks OX40 (CD134)	10 mg/kg intravenous GBR 830 on day 1 (baseline) and day 29	improvement in EASI; significant progressive reductions in TH1 (IFN-γ/CXCL10), TH2 (IL-31/CCL11/CCL17), and TH17/TH22 (IL-23p19/IL-8/S100A12) mRNA expression in lesional skin; higher reductions in hyperplasia measures than placebo	[158]

DLQI—Dermatology Life Quality Index; SCORAD—Scoring Atopic Dermatitis; POEM—Patient-Oriented Eczema Measure; EASI-75—a 75% reduction from baseline in the Eczema Area and Severity Index; EASI-50—a 50% reduction from baseline in the Eczema Area and Severity Index; TCS—topical corticosteroids.
